# The Outcome of Hospital-Acquired Bloodstream Infection and Its Associated Factors in Critical Care Unit

**DOI:** 10.21315/mjms2024.31.6.13

**Published:** 2024-12-31

**Authors:** Nurul Rahatul Ilyani Mohamed Shukri, Shamsul Kamalrujan Hassan, Siti Suraiya Md Noor, Siti Azrin Ab Hamid, Nik Abdullah Nik Mohamad, Wan Fadzlina Wan Muhd Shukeri, Mohd Zulfakar Mazlan

**Affiliations:** 1 Department of Anaesthesiology and Intensive Care, School of Medical Sciences, Universiti Sains Malaysia, Kelantan, Malaysia; 2 Hospital Pakar Universiti Sains Malaysia, Kelantan, Malaysia; 3 Department Microbiology and Parasitology, School of Medical Sciences, Universiti Sains Malaysia, Kelantan, Malaysia; 4 Biostatistics and Research Methodology Unit, School of Medical Sciences, Universiti Sains Malaysia, Kelantan, Malaysia

**Keywords:** hospital-acquired bloodstream infection (BSI), treatment failure, outcome, multidrug-resistant (MDR), extensively drug resistant (XDR)

## Abstract

**Background:**

Hospital-acquired bloodstream infections (BSI) are associated with high morbidity and mortality rates. This study was conducted to describe the outcomes and the prognosis of hospital-acquired BSI in the Critical Care Unit, Hospital Pakar Universiti Sains Malaysia (HPUSM), as well as to identify associated factors of treatment failure and mortality at 28 days.

**Methods:**

This prospective cohort study was conducted in the Critical Care Unit of HPUSM from September 2019 to March 2021. Eligible participants included patients with a positive blood culture recorded after 48 hours of admission to hospital.

**Results:**

There was a total of 250 patients, whose positive blood cultures were isolated. The main isolated organisms were *Klebsiella pneumonia* (23.6%), *Pseudomonas* spp. (19.2%), *Escherichia coli* (12.8%) and *Acinetobacter sp.* (9.2%). The mortality of hospital-acquired BSI was 27.6%. Multiple logistic regression analysis revealed that age [adjusted odds ratio (OR) = 1.06; 95% confidence interval (CI): 1.03, 1.09; *p* < 0.001], cases with extended-spectrum beta-lactamases (ESBL) (adjusted OR = 5.57; 95% CI: 2.04, 15.21; *p* = 0.001), with multidrug-resistant (MDR) organisms (adjusted OR = 14.70; 95% CI: 3.97, 54.48; *p* < 0.001) and those with a sequential organ failure assessment (SOFA) score > 11 (adjusted OR = 4.16; 95% CI: 1.31, 13.19; *p* = 0.015) had statistically significant associations with treatment failure. Factors significantly associated with 28-day mortality included age (adjusted OR: 1.06: 95% CI; 1.03, 1.09; *p* < 0.001), MDR organisms (adjusted OR = 14.70; 95% CI: 3.97, 54.48; *p* < 0.001) and SOFA score > 11 (adjusted OR = 4.16; 95% CI: 1.31, 13.19; *p* = 0.015).

**Conclusion:**

The elderly, ESBL, MDR organisms and high SOFA scores were associated with treatment failure and 28-day mortality in hospital-acquired BSI.

## Introduction

Sepsis has been the leading cause of death worldwide for several decades (1). Despite innovative approaches in its management, sepsis remains a substantial burden on healthcare systems (2). Left untreated, it not only escalates mortality rates but also poses the risk of irreversible multi-organ dysfunction (3). The escalating morbidity often necessitates multi-organ support, consequently increasing the cost of managing sepsis patients (2–6). Among the myriad causes of sepsis, bloodstream infections (BSI) stand out as a major contributing factor (2).

Hospital-acquired infections pose a significant threat to life, particularly when involving the bloodstream and lungs (2). According to the Centre for Disease Control in 1998, hospital-acquired BSIs are defined as positive blood cultures isolated 48 hours after hospital admission that were not present upon admission (7). Brun-Buisson et al. (8) reported an alarming statistic, estimating that approximately 250,000 Americans suffer from hospital-acquired BSIs annually. In another study by Sagana and Hyzy (9), the incidence was reported at 0.6%, constituting one-tenth of hospital-acquired infections.

There is compelling evidence pointing to the substantial impact of intensive care unit (ICU) BSI on morbidity and mortality among critically ill patients (10, 11). The occurrence rate of ICU BSI, ranging from 5% to 10%, has been reported in various epidemiological studies (4, 8, 12, 13). Moreover, these infections carry a significant mortality risk, with rates reported as high as 30.0% to 42.6% according to studies by Garrouste-Orgeas et al. and Prowle et al., respectively (14, 15).

Critically ill patients are vulnerable to developing hospital-acquired BSI as they face higher risk factors, such as multiple comorbidities, severe acute illness and the use of invasive devices (16). Although hospital-acquired BSIs come from multiple sources, the attributable mortality rate is higher if isolated from the lung, catheter-related and abdominal source (14, 15, 17). The case-fatality rate of catheter-related BSI (CRBSI) reported internationally is 12% to 25% (18, 19). In the US, CRBSI can reach up to 28,000 deaths per year and cost USD2.3 billion annually (9). However, the mortality rates do not differ between antibiotic-resistant organisms and antibiotic-sensitive organisms (16).

Recently, rapid changes in epidemiological data have been extensively reported worldwide, particularly with the emergence of drug resistant microorganisms (20–22). As such, they constitute an additional burden for healthcare systems in both developed and developing countries (17, 22). The most common pathogen that caused nosocomial infection, as revealed in the EUROBACT study in 2012, was gram-negative bacilli, including members of the *Enterobacteriaceae* family, such as *Klebsiella pneumonia* and *Escherichia coli* and non-lactose fermenters, such as *Pseudomonas* spp. and *Acinetobacter* spp. (17). The global pandemic of antimicrobial resistance among *Enterobacteriaceae* in the past two decades has been, in large part, caused by the emergence and dissemination of extended-spectrum betalactamases (ESBL) and carbapenemases in these organisms (20).

Despite extensive data reporting on the characteristics, determinants and associated factors in the treatment failure of hospital-acquired BSI globally (14, 23), there remains a paucity of information, particularly on ICU patients in developing countries (24). This gap is particularly pronounced in the tertiary teaching university hospitals in the Asia-Pacific region. Hospital-acquired BSI is widely acknowledged as a significant patient safety concern and a key marker of care quality (23, 25). Consequently, this study aims to fill this gap by identifying the outcomes of hospital-acquired BSI and its associated factors within the critical care unit.

## Methodology

**Figure 1:**
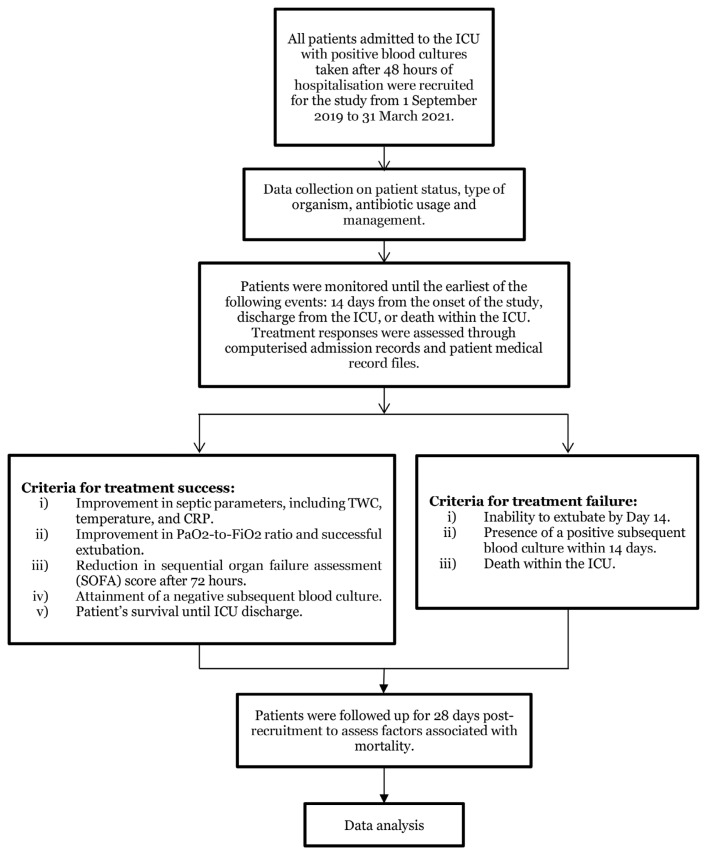
Study flow chart

This was a prospective cohort study conducted in the critical care unit in Hospital Pakar Universiti Sains Malaysia (HPUSM), involving the Medical, Surgical and Trauma ICUs and the Surgical and Medical High-dependency units, from September 2019 to March 2021. The ICU admits an average of 1,600 patients annually. Based on a previous study and using a two-proportion formula with a 95% confidence interval (CI) and 80% power, while accounting for a 20% drop-out rate, the necessary sample size was determined to be 50 patients per group (26). The patients with a diagnosis of BSI during hospitalisation and requiring admission to the critical care unit for the appropriate management or patients already in ICU who developed BSI after 48 hours of admission were enrolled (Figure 1). The study only focuses on the first episode of blood culture positivity. The exclusion criteria were blood culture reported as contaminated by the microbiology laboratory and previous inclusion.

Upon the electronic identification of positive blood cultures, eligible patients were enrolled in the study. Comprehensive data were extracted from medical records using a data collection form encompassing clinical, biological and microbiological information, as well as patient outcomes. The collected data for each patient included demographic details (age, gender, weight, height, and admission source), dates of the first positive blood culture collection and its positivity, source of infection, presence of sepsis, severity of illness, comorbidities and details of management, including source control and antimicrobial drugs. All study-related data were exclusively sourced from patient medical records and ICU charts, and no additional tests were conducted for the purpose of this study.

### Study Operational Data Collection Procedures

All patients were closely monitored until day 14, during which they were categorised into either the treatment success or treatment failure groups. Treatment success was defined as patients meeting any of the following criteria: improvement in septic parameters [temperature normalisation/reduced total white cell count (TWC)/reduced C-reactive protein (CRP)/ procalcitonin levels], successful extubation without reintubation within 24 hours, a decrease in the sequential organ failure assessment (SOFA) score after 72 hours, negative subsequent cultures within 14 days or being alive upon ICU discharge. On the other hand, patients were considered to have treatment failure if they exhibited persistent or worsening signs of infection (e.g., fever, increasing TWC, CRP or procalcitonin levels), persistent infection at the source, inability to extubate on day 14 or death in the ICU. Follow-up continued until day 28 from the onset of BSI (Figure 1). Survival status, as well as any relapses or new episodes of BSI, were meticulously recorded until ICU discharge. All-cause mortality within 28 days since the detection of the first positive blood culture was also determined.

### Data Analysis

Data entry and analysis were conducted using Statistical Package for the Social Sciences (SPSS) version 27 (IBM Corp., Armonk, NY, US). Descriptive statistics were employed to summarise the characteristics of subjects. Numerical data were presented as the mean and standard deviation (SD) or median and interquartile range (IQR) based on their normal distribution. Association between categorical data were analysed by Pearson chi-square or Fisher Exact test. The significance level was set at *p* < 0.05.

Univariate analyses of associated factors for treatment failure and 28-day mortality were performed using simple logistic regression (SLR). All variables with *p* < 0.25 and that were biologically or clinically relevant according to SLR were included in multiple logistic regression. Variable selection was carried out using forward, backward, forward stepwise, and backward stepwise selections. The model with forward stepwise selection was ultimately chosen. Crude and adjusted odds ratios (OR) with 95% CI were computed for variables associated with treatment failure and mortality. The significance level was set at *p* < 0.05.

## Results

A total of 250 patients were included in the study, and their baseline demographic characteristics are presented in Table 1. The mean (SD) age of the patients was 57.35 (14.12) years, with 60.8% being male. Among them, 155 (62%) patients acquired BSI in the ICU, while 95 (38%) patients contracted infections in the ward. The majority of BSI cases developed after 72 hours of hospitalisation. The study found that 179 patients (71.6%) achieved treatment success, whereas 71 patients (28.4%) experienced treatment failure (Table 1).

Many patients presented with multiple underlying illnesses, including diabetes mellitus (74.0%), hypertension (70.8%), chronic kidney disease (CKD) (28.8%), ischaemic heart disease (IHD) (8.4%), cerebral vascular accident (CVA) (7.2%), and malignancy (8.0%) (Table 1). There were statistically significant associations between treatment failure and CKD, CVA and other medical conditions such as liver cirrhosis (*p* = 0.001, 0.035 and 0.048, respectively). Interestingly, diabetes and IHD did not show a significant association with treatment failure.

The acute severity score was lower in the treatment success group, with an APACHE score < 25 observed in 89.8% of cases. The SOFA score also demonstrated an association with response to treatment (*p* < 0.001). Specifically, 73.2% of treatment failures exhibited a SOFA score greater than 11. Urinary and infected lines were less likely, while respiratory sources were more likely in treatment failure compared to the treatment success group (Table 1).

The majority of patients (about 88%) exhibited the isolation of a single organism, showing a significant association (*p* < 0.005) with treatment response (Table 2). The predominant organisms identified in this study were mainly gram-negative bacteria, constituting 76.8%, with the *Enterobacteriaceae* group, *K. pneumonia*, *E. coli* and non-fermenter gram-negative bacilli such as *Pseudomonas* spp. and *Acinetobacter* spp. Gram-positive bacteria accounted for 16.0%, while fungi were the least prevalent at 7.2%. Notably, *E. coli* and *Acinetobacter* organisms were associated with treatment failure. Furthermore, susceptible strain (SUS) organisms exhibited a statistically significant association with treatment success compared to resistant strains [ESBL, multidrug-resistant (MDR) and extensively drug resistant (XDR)] (*p* < 0.001).

In the univariate analysis (Table 3), the statistically significant variables were age (*p* = 0.016), gender (*p* = 0.012), CKD (*p* = 0.001), CVA (*p* = 0.041), APACHE score (*p* = 0.001), and SOFA score (*p* < 0.001). In the multivariate analysis (Table 4), older patients had 6% higher odds of experiencing treatment failure *p* < 0.001. BSI patients with an MDR strain increase the probability of treatment failure by 14.70 compared to those with SUS strain (*p* < 0.001). BSI patients with XDR strain increase the likelihood of treatment failure by 4.24 compared to those with SUS strain (*p* = 0.039).

In terms of the outcome of BSI in this study, 181 patients (72.6%) were alive, while 69 patients (27.4%) had succumbed by the 28th day of follow-up. The majority of fatalities occurred in the ICU (58 patients), with an additional 11 deaths in the ward due to other causes. Notably, 47 patients (68.1%) who died had acquired BSI in the ICU. Epidemiologic data on the outcome are presented in Table 5.

The mean age of patients with BSI was 57.35 (SD = 14.12) years, and older patients had a poorer outcome, with a mean age of 63.48 (SD = 11.99) years. Among the deceased, a significant proportion had multiple comorbidities, including 58 patients (84.1%) with diabetes mellitus, 58 patients (84.1%) with hypertension, and 29 patients (42.0%) with CKD (*p* = 0.025, 0.004, and 0.004, respectively). A majority of patients (84.8%) with an APACHE score of less than 25 had a better outcome. The presence of ESBL and MDR strains was higher in the group that did not survive, with 13 and 12 patients, respectively.

In the univariate analysis, as presented in Table 6, several variables demonstrated statistical significance, including age (*p* < 0.001), diabetes mellitus (*p* = 0.028), hypertension (*p* = 0.006), CKD (*p* = 0.005), chronic obstructive pulmonary disease (*p* = 0.033), other diseases such as liver disease (*p* = 0.046), APACHE score > 25 (*p* < 0.001), and SOFA score > 11 (*p* < 0.001).

In the multivariate analysis, the variables found to be statistically significant were age (*p* < 0.001) and SOFA score > 11 (*p* = 0.015) (Table 7). For every one-unit increase in age among patients with BSI, probability of mortality from the infection increased by 6% (adjusted OR = 1.06; 95% CI: 1.03, 1.09; *p* < 0.001). Patients with ESBL, MDR, and XDR strains had 5.57, 14.70, and 4.24-times higher likelihood of mortality, respectively, compared to those with SUS. BSI patients with a SOFA score > 11 had 4.16 times higher odds of mortality compared to BSI patients with a SOFA score < 9 (adjusted OR = 4.16; 95% CI: 1.31, 13.19; *p* = 0.015).

## Discussion

In this study, age, microbiologically resistant organisms, and a high SOFA score were revealed as independent factors associated with treatment failure and mortality in hospital-acquired BSI. The results indicated that higher age increased the likelihood of experiencing treatment failure. This finding aligns with the EUROBACT study in 2012, which reported a similar association (17). The study discovered that patients over 70 years old had a higher risk of developing healthcare-associated BSI, with an odds ratio of 2.86 (95% CI: 1.31–6.25) (27). Another study highlighted that adults aged 65 years old and above face the highest risk of morbidity and mortality from BSI, with case-fatality rates rising with age: 17.3% (65–74 years), 24.6% (75–84 years), and 26.9% (85 years and above) (28).

Notably, microbiological data played a significant role in influencing the outcome of BSI, surpassing the impact of patient age and severity scores. The incidence of CKD and end-stage renal failure has doubled over the past two decades (29). These populations are more susceptible to developing BSIs, which are often attributed to factors such as catheter-related infections, an immunocompromised state, malnutrition and multiple comorbidities, including diabetes, which further impairs immune function (29, 30), increasing vulnerability to difficult-to-treat infections such as ESBL, MDR, and XDR organisms. The EUROBACT-2 study identified pneumonia and intravascular catheters as common BSI sources, with gram-negative bacteria being prevalent, notably *Klebsiella*, *Acinetobacter*, *E. coli*, and *Pseudomonas*, mirroring this present study’s findings (31).

The elevated urea concentration in CKD leads to alterations in intestinal flora, increasing the production of gut-derived toxins and compromising the intestinal epithelial barrier. This, in turn, heightens the risk of bacterial translocation into the bloodstream (30). In this study, patients with CKD had no association with treatment failure compared to those without CKD. Tabah et al. (17) demonstrated that renal disease had an insignificant association with the outcome of BSI.

The altered pharmacokinetics and pharmacodynamics of antimicrobial therapy in critically ill patients have become a challenge for clinicians, particularly concerning the issue of under-dosing (32–34). A comprehensive review by Roberts and Lipman suggests that individualised dosing regimens, utilising therapeutic drug monitoring, can assist clinicians in providing optimal care for patients with impaired renal function (32), ultimately improving the outcomes of ICU patients.

In this study’s findings, patients with SOFA score > 11 had an association with treatment failure compared to patients with SOFA score < 9. This was aligned with several studies performed in various regions (17, 22, 30, 31, 35). Some data proposed that treatment failure is more complicated for more severely ill patients and that this is related to an increased incidence of microorganisms such as methicillin-resistant *Staphylococcus aureus* (MRSA), *Pseudomonas aeruginosa* and *Enterobacteriaceae* harbouring ESBL (35). In China, Sun (36) reported in a multivariate logistic regression analysis for death that a SOFA score > 7 was significantly associated with 28-day mortality.

After reviewing the literature, most of the studies reported that additional factors that independently influenced the outcome of BSI were multiple occurrences of infection and ICU admission for treatment of sepsis (37, 38). A landmark trial from the EUROBACT study in 2012 reported that an increased 28-day mortality was observed among patients who had hospital-acquired bacteraemia and were admitted to ICU (adjusted OR = 1.56; 95% CI 1.04–2.3; *p* = 0.03) (17). The EUROBACT-2 study published in 2023 revealed there was an association of 28 days of mortality (31).

In the present study, it is concluded that about 88% of the BSIs are due to monomicrobial organisms as compared to 12% caused by polymicrobial organisms. This study found that patients with monomicrobial causative agents had higher treatment success compared to polymicrobial agents. However, the outcome of this phenomenon was not statistically significant. There must be some other contributing factors that independently increase the risk of mortality regardless of the number of organisms isolated, such as multiple comorbidity, the severity of SOFA score and resistant organism patterns. This finding is inconsistent with other population-based studies (17, 39–41). Pittet et al. reported a more direct relationship between increased mortality rate and polymicrobial than with monomicrobial in all series after controlling for other confounders (40). The researchers revealed that the family of *Enterobacteriaceae*, nongroup A *Streptococci* and *Pseudomonas* spp. were extremely common in polymicrobial bacteraemia. Deaths related to polymicrobial bacteraemia were two times higher than monomicrobial. The factors associated with increased mortality included age greater than 40 years old, neutropenia and inadequate antimicrobial therapy (40).

Previous studies have demonstrated an important association between antibiotic resistance and adverse outcomes for patients with gram-negative bacteraemia (37, 42, 43). However, causality remains unclear. Some proposed that ICU patients were exposed to extensively broad-spectrum antibiotic usage and prolonged ICU stays (44). Thus, this condition will cause the gastrointestinal bacterial normal flora to mutate and become antibiotic-resistant microorganisms (44). With the rapid emergence of resistant superbugs, this has become a major threat to our healthcare systems. Hence, it is very important to identify the epidemiology of microorganism patterns in local ICU before initiating antibiotic treatment (45, 46).

Antimicrobial resistance increases morbidity, mortality, length of hospital stay and healthcare costs (47–49). The emergence of MRSA among gram-positive bacteria, *S. aureus* and MDR, ESBL-producing bacteria and XDR organisms among gram-negative bacteria have become a major global healthcare threat in the 21st century (47). Balkan et al. (50) found that carbapenem-resistant *Enterobacteriaceae* (CRE) is the culprit for BSI, thus, posing a tremendous challenge for treating clinicians across university hospital in Istanbul. To reverse antimicrobial resistance back towards a state of susceptibility is unfeasible. To kerb this crisis, the application of novel strategies is necessary, such as using combinations of antimicrobial drugs that counteract the mechanisms of antibiotic resistance expressed by the pathogen (20, 47). In the present study, the findings are consistent with recent epidemiology data, as ESBL, MDR followed by XDR isolated pathogens were associated with increasing the odds of treatment failure. The possible reason could be isolated resistant organisms associated with prolonged antibiotic inadequacy as revealed by Tabah et al. (17).

Furthermore, this present study revealed that individuals with XDR strains had higher odds of mortality from infection than those with SUS strains. Surprisingly, this contradicts findings from the EUROBACT study in 2022, where XDR resistance levels were not associated with higher 28-day mortality when compared with MDR strains (17). While the issue remains controversial, experimental studies postulate that resistance to antimicrobial agents may be linked to decreases in bacterial fitness, metabolic activity (51) or virulence (52).

The influence of antimicrobial treatment in MDR BSI deserves some comment. Previous antibiotic exposure information history of MDR carriers are major determinants of first choice antimicrobials in nosocomial BSI. An initial dose of antimicrobial should be based on the pharmacokinetic profile (53). Generally, a high dose is recommended at the initiation of treatment (54, 55). Insufficient knowledge of antimicrobial pharmacokinetics and pharmacodynamics is a common factor causing antibiotic failure particularly in critically ill patients and was extensively reviewed by Roberts and Lipman (32). In this subpopulation, low plasma concentrations of antimicrobials were responsible for antibiotic failure because of an increased volume of distribution that follows the increase in capillary permeability. Eventually, trials to tackle under-dosing may be offset by renal or hepatic dysfunction (31). A large cohort study by Kumar et al. (56) stated that combination antibiotics are recommended for severe infections and have been proven to improve survival. For CRE infections, for example, double colistin-based combinations, sometimes triple, were advocated as they were associated with significantly better outcomes when compared to non-colistin-based regimens (50). The combinations are mandated due to an increase in the spectrum of treatment when MDR is suspected. In most cases, the combination should be pursued for no more than two to five days (53).

## Limitations

Firstly, it is important to note that this study was conducted in a single-centred tertiary hospital, limiting its generalisability to the overall population. Secondly, a larger sample size would be beneficial to reduce bias and enhance the study’s power, allowing for better representation of the entire ICU patient population. The larger 95% CI results should be interpreted cautiously due to the relatively small sample size. However, this study’s results on the organisms causing BSI were consistent with the EUROBACT-2 study, where the four most common organisms were *Klebsiella* spp. (27.9%), *Acinetobacter* spp. (20.3%), *E. coli* (15.8%), and *Pseudomonas* spp. (14.3%) (56). The results also indicate a 27.6% mortality rate compared to the EUROBACT-2 study, which reported a 37.1% of the 28-day mortality (56). Additionally, the study’s duration was relatively short, spanning only one year, and extending the data collection period could provide more valuable insights. Finally, the relative heterogeneity of patients in this study, spanning various critical care units in HPUSM, poses a limitation. For instance, medical patients had higher sepsis scores than neurosurgical patients, although this factor did not influence the predictors for treatment failure and the outcome of BSI.

## Conclusion

In conclusion, the mortality rate for hospital-acquired BSI in this study was 27.6%. The factors associated with treatment failure and 28-day mortality were old age, infection with resistant strain microorganisms (ESBL, MDR, and XDR) and higher SOFA scores.

## Figures and Tables

**Table 1:** The baseline characteristics of patients with hospital-acquired BSI in HPUSM based on the responses to the treatment status (n = 250)

Variable	Success (n = 179) n (%)	Failure (n = 71) n (%)	Total n (%)	*p*-value
Age (years)*	55.98 (13.71)	60.80 (14.64)	57.35 (14.12)	0.015^a^

Gender				0.014^b^
Male	100 (55.9)	52 (73.2)	152 (60.8)	
Female	79 (44.1)	19 (26.8)	98 (39.2)	

Onset				0.101^b^
< 3 days	10 (5.6)	2 (2.8)	12 (4.8)	
3–7 days	113 (63.1)	37 (52.1)	15 (60.)	
> 7 days	56 (31.3)	32 (45.1)	88 (35.2)	

Admission				0.150^b^
Ward	73 (40.8)	22 (31.0)	95 (38.0)	
ICU	106 (59.2)	49 (69.0)	155 (62.0)	

Department				0.267^c^
Medical	80 (44.7)	32 (45.1)	112 (44.8)	
Surgical	49 (27.4)	28 (39.4)	77 (30.8)	
Orthopaedics	10 (5.6)	2 (2.8)	12 (4.8)	
Neurology	12 (6.7)	4 (5.6)	16 (6.4)	
Cardiology	1 (0.6)	1 (1.4)	2 (0.8)	
Trauma	21 (11.7)	3 (4.2)	24 (9.6)	
Obstetrics and gynaecology	6 (3.4)	1 (1.4)	7 (2.8)	

APACHE score				0.001
< 25	161 (89.9)	51 (71.8)	212 (84.8)	
25–35	15 (8.4)	15 (21.1)	30 (12.0)	
> 35	3 (1.7)	5 (7.0)	8 (3.2)	

SOFA score				< 0.001^b^
< 9	45 (25.1)	4 (5.6)	49 (19.6)	
9–11	67 (37.4)	15 (21.1)	82 (32.8)	
> 11	67 (37.4)	52 (73.2)	119 (47.6)	

Diabetes mellitus				0.154^b^
Yes	128 (71.5)	57 (80.3)	186 (74.0)	
No	51 (28.5)	14 (19.7)	65 (26.0)	

Hypertension				0.077 b
Yes	121 (67.6)	56 (78.9)	177 (70.8)	
No	58 (32.4)	15 (21.1)	73 (29.2)	

CKD				0.001 b
Yes	41 (22.9)	31 (43.7)	72 (28.8)	
No	138 (77.1)	40 (56.3)	178 (71.2)	

COPD				0.306 b
Yes	11 (6.1)	7 (9.9)	18 (7.2)	
No	168 (93.9)	64 (90.1)	232 (93.2)	

IHD				0.985 b
Yes	15 (8.4)	6 (8.5)	21 (8.4)	
No	164 (91.6)	65 (91.5)	229 (91.6)	

CVA				0.035 b
Yes	9 (5.0)	9 (12.7)	18 (7.2)	
No	170 (95.0)	62 (87.3)	232 (92.8)	

Malignancy				0.086 b
Yes	11 (6.1)	9 (12.7)	20 (8.0)	
No	168 (93.9)	62 (87.3)	230 (92.0)	

Others				0.048 b
Yes	8 (4.5)	8 (11.3)	16 (6.4)	
No	171 (95.5)	63 (88.7)	234 (93.6)	

Notes:

* mean (SD);

a Independent *t*-test applied; normality and equal variance assumptions met;

b Pearson chi-square applied;

c Fisher exact test applied;

COPD = Chronic Obstructive Pulmonary Disease

**Table 2:** The clinical characteristics of patients with hospital-acquired BSI (n = 250)

Variable	Success (n = 179) n (%)	Failure (n = 71) n (%)	Total n (%)	*p*-value
Gram stain				0.867^a^
Positive	30 (16.8)	10 (14.1)	40 (16.0)	
Negative	136 (76.0)	56 (78.9)	192 (76.8)	
Fungal	13 (7.3)	5 (7.0)	18 (7.2)	

Organism 1				0.083^b^
*K. pneumonia*	48 (26.8)	11 (15.5)	59 (23.6)	
*Pseudomonas* sp	36 (20.1)	12 (16.9)	48 (19.2)	
MRSA	10 (5.6)	2 (2.8)	12 (4.8)	
MSSA	14 (7.8)	3 (4.2)	17 (6.8)	
*Acinetobacter* sp	11 (6.1)	12 (16.9)	23 (9.2)	
*Candida albicans*	5 (2.8)	2 (2.8)	7 (2.8)	
Non-albicans	8 (4.5)	4 (5.6)	12 (4.8)	
Others	7 (3.9)	4 (5.6)	11 (4.4)	
*E. coli*	22 (12.3)	10 (14.1)	32 (12.8)	
*Stenotrophomonas maltophilia*	5 (2.8)	7 (9.9)	12 (4.8)	
*Enterobacter aerogenes*	8 (4.5)	4 (5.6)	12 (4.8)	
Coagulase negative staphaureus	1 (0.6)	0 (0.0)	1 (0.4)	
*Enterococcus faecium*	4 (2.2)	0 (0.0)	4 (1.6)	

Organism 2				0.008^b^
*K. pneumonia*	2 (1.1)	2(2.8)	4 (1.6)	
*Pseudomonas aeroginosa*	6 (3.4)	1 (1.4)	7 (2.8)	
MSSA	1 (0.6)	0 (0.0)	1 (0.4)	
*Acinetobacter* sp	1 (0.6)	3 (4.2)	4 (1.6)	
*C. albican*	2 (1.1)	0 (0.0)	2 (0.8)	
Non-albican	2 (1.1)	1 (1.4)	3 (1.2)	
*E. coli*	1 (0.6)	5 (7.0)	6 (2.4)	
*S. maltophilia*	0 (0.0)	1 (1.4)	1 (0.4)	
*E. aerogenes*	1 (0.6)	1 (1.4)	2 (0.8)	
No	163 (91.1)	57 (80.3)	220 (88.0)	

Strain				< 0.001^b^
Sensitive	152(84.9)	31 (43.7)	184 (73.2)	
ESBL	7 (3.9)	15 (21.1)	22 (8.8)	
MDR	5 (2.8)	12 (16.9)	17 (6.8)	
XDR	4 (2.2)	9 (12.7)	13 (5.2)	
CRE	1 (0.6)	3 (4.2)	4 (1.6)	
MRSA	10 (5.6)	1 (1.4)	11 (4.4)	

Notes:

a Pearson chi-square applied;

b Fisher exact test applied;

MRSA = methicillin-resistant *Staphylococcus aureus*; CRE = carbapenem-resistant *Enterobacteriaceae*; MSSA = methicillin-sensitive *Staphylococcus aureus*

**Table 3:** The baseline characteristics associated with the respond status towards treatment in patients with hospital-acquired BSI admitted to HPUSM using SLR (n = 250)

Variables	b	Crude OR (95% CI)	Wald statistics	*p*-value
Age (years)	0.03	1.03 (1.01, 1.05)	5.78	0.016

Gender				
Female	0	1		
Male	1	2.16 (1.18, 3.95)	6.29	0.012

Weight (kg)	−0.01	0.99 (0.97, 1.01)	0.88	0.349

Height (cm)	0.02	1.02 (0.98, 1.07)	0.71	0.398

Onset				
< 3 days	0	1		
3–7 days	0.49	1.64 (0.34, 7.81)	0.38	0.536
> 7 days	1.05	2.86 (0.59, 13.86)	1.70	0.193

Admission				
Ward	0	1		
ICU	0.43	1.53 (0.86, 2.75)	2.06	0.152

Department				
Medical	0	1		
Surgical	−0.05	0.95 (0.55, 1.65)	0.03	0.861

APACHE score				
< 2*5*	0	1		
> 25	1.26	3.51 (1.72, 7.14)	11.99	0.001

SOFA score				
< 9	0	1		
9–11	0.924	2.52 (0.78, 8.08)	2.41	0.120
> 11	2.167	8.73 (2.95, 25.84)	15.33	< 0.001

Diabetes mellitus				
No	0	1		
Yes	0.484	1.62 (0.83, 3.17)	2.01	0.156

Hypertension				
No	0	1		
Yes	0.582	1.79 (0.93, 3.43)	3.078	0.079

CKD				
No	0	1		
Yes	0.959	2.61 (1.45, 4.68)	10.34	0.001

COPD				
No	0	1		
Yes	0.513	1.67 (0.62, 4.50)	1.03	0.310

IHD				
No	0	1		
Yes	0.009	1.01 (0.38, 2.71)	0.00	> 0.950

CVA				
No	0	1		
Yes	1.009	2.74 (1.04, 7.22)	4.17	0.041

Malignancy				
No	0	1		
Yes	0.80	2.22 (0.88, 5.61)	2.83	0.093

Others				
No	0	1		
Yes	0.10	2.71 (0.98, 7.54)	3.67	0.055

Gram stain				
Negative	0	1		
Positive	−0.21	0.81 (0.37, 1.77)	0.282	0.596
Fungal	−0.07	0.93 (0.32, 2.74)	0.015	0.901

Strain				
Sensitive	0	1		
ESBL	2.35	10.52 (3.96, 27.91)	22.27	< 0.001
MDR	2.47	11.77 (3.87, 35.80)	18.87	< 0.001
XDR	2.40	11.03 (3.19, 38.11)	14.41	< 0.001
CRE	2.69	14.71 (1.48, 146.12)	5.27	0.022
MRSA	−0.71	0.49 (0.06, 3.97)	0.45	0.504

Note: MRSA = methicillin-resistant *S. aureus*; CRE = carbapenem-resistant *Enterobacteriaceae*

**Table 4:** Associated factors of treatment failure in patients with hospital-acquired BSI admitted to HPUSM using multiple logistic regression (n = 250)

Variables	b	Adjusted OR (95% CI)	Wald statistics	*p*-value
Age (years)	0.06	1.06 (1.03, 1.09)	14.11	< 0.001

Other				
No	0	1		
Yes	1.50	4.47 (1.32, 15.17)	5.76	0.016

Strain				
Sensitive	0	1		
ESBL	1.72	5.57 (2.04, 15.21)	11.24	0.001
MDR	2.69	14.70 (3.97, 54.48)	16.18	< 0.001
XDR	1.44	4.24 (1.08, 16.71)	4.26	0.039
CRE	22.42	−0.59 (0.07, 5.08)	−0.24	> 0.950
MRSA	−0.53			0.628

SOFA score				
< 9	0	1		
9–11	0.49	1.63 (0.48, 5.57)	0.61	0.436
> 11	1.43	4.16 (1.31, 13.19)	5.87	0.015

Notes: Forward LR Multiple Logistic Regression model was applied. Multicollinearity and interaction term were checked and not found. Hosmer-Lemeshow test (*p* = 0.390), classification table (overall correctly classified percentage = 81.2%), and area under the Receiver Operating Characteristics curve (63.1%) were applied to check the model fit. MRSA = methicillin-resistant *S. aureus*; CRE = carbapenem-resistant *Enterobacteriaceae*

**Table 5:** The baseline characteristics of patients with hospital-acquired BSI in HPUSM based on the survival status (n = 250)

Variable	Alive (n = 181) n (%)	Death (n = 69) n (%)	Total n (%)	*p*-value
Age (years)*	55.01 (14.20)	63.48 (11.99)	57.35 (14.12)	< 0.001^a^

Gender				0.080^b^
Male	104 (57.5)	48 (69.6)	152 (60.8)	
Female	77 (42.5)	21 (30.4)	98 (39.2)	

Onset				0.606^b^
< 3 days	8 (4.4)	4 (5.8)	12 (4.8)	
3–7 days	112 (61.9)	38 (55.1)	150 (60.0)	
> 7 days	61 (33.7)	27 (39.1)	88 (35.2)	

Admission				0.219^b^
Ward	73 (40.3)	22 (31.9)	95 (38.0)	
ICU	108 (59.7)	47 (68.1)	155 (62.0)	

Department				0.038^c^
Medical	78 (43.1)	34 (49.3)	112 (44.8)	
Surgical	50 (27.6)	27 (39.1)	77 (30.8)	
Orthopaedics	9 (5.0)	3 (4.3)	12 (4.8)	
Neurology	15 (8.3)	1 (1.4)	16 (6.4)	
Cardiology	1 (0.6)	1 (1.4)	2 (0.8)	
Trauma	22 (12.2)	2 (2.9)	24 (9.6)	
Obstetrics and gynaecology	63 (3.3)	1 (1.4)	7 (2.8)	

APACHE score				0.001
< 25	163 (90.1)	49 (71.0)	212 (84.8)	
25–35	14 (7.7)	16 (23.2)	30 (12.0)	
> 35	4 (2.2)	4 (5.8)	8 (3.2)	

SOFA score				< 0.001
< 9	44 (24.3)	5 (7.2)	49 (19.6)	
9–11	67 (37.0)	15 (21.7)	82 (32.8)	
> 11	70 (38.7)	49 (71.0)	119 (47.6)	

Source				0.307^b^
Primary (blood)	60 (33.1)	20 (29.0)	80 (32.0)	
Respiratory	71 (39.2)	30 (43.5)	101 (40.4)	
Intraabdominal	22 (12.2)	14 (20.3)	36 (14.4)	
Genitourinary tract	12 (6.6)	1 (1.4)	13 (5.2)	
Bone and soft tissues	13 (7.2)	3 (4.3)	16 (6.4)	
Catheter-related	3 (1.7)	1 (1.4)	4 (1.6)	

Organism				0.151^b^
*K. pneumonia*	47 (26.0)	12 (17.4)	59 (23.6)	
*Pseudomonas* sp	34 (18.8)	14 (20.3)	48 (19.2)	
MRSA	10 (5.5)	2 (2.9)	12 (4.8)	
MSSA	16 (8.8)	1 (1.4)	17 (6.8)	
*Acinetobacter* sp	12 (6.6)	11 (15.9)	23 (9.2)	
*C. albicans*	6 (3.3)	1 (1.4)	7 (2.8)	
Non-albicans	8 (4.4)	4 (5.8)	12 (4.8)	
Others	6 (3.3)	5 (7.2)	11 (4.4)	
*E. coli*	24 (13.3)	8 (11.6)	32 (12.8)	
*S. maltophilia*	5 (2.8)	7 (10.1)	12 (4.8)	
*E. aerogenes*	8 (4.4)	4 (5.8)	12 (4.8)	
Coagulase negative staph aureus	1 (0.6)	0 (0.0)	1 (0.4)	
*E. faecium*	4 (2.2)	0 (0.0)	4 (1.6)	

Organism 2				0.049^b^
*K. pneumonia*	3 (1.7)	1 (1.4)	4 (1.6)	
*P. aeroginosa*	6 (3.3)	1 (1.4)	7 (2.8)	
MSSA	1 (0.6)	0 (0.0)	1 (0.4)	
*Acinetobacter* sp	1 (0.6)	3 (4.3)	4 (1.6)	
*C. albican*	2 (1.1)	0 (0.0)	2 (0.8)	
Non albican	2 (1.1)	1 (1.4)	3 (1.2)	
*E. coli*	3 (1.7)	3 (4.3)	6 (2.4)	
*S. maltophilia*	0 (0.0)	1 (1.4)	1 (0.4)	
*E. aerogenes*	0 (0.0)	2 (2.9)	2 (0.8)	
No	163 (74.1)	57 (25.9)	220 (88.0)	

Gram stain				0.601
Positive	31 (17.1)	9 (13.0)	40 (16.0)	
Negative	136 (75.1)	56 (81.2)	192 (76.8)	
Fungal	14 (7.7)	4 (5.8)	18 (7.2)	

Strain				< 0.001^b^
Sensitive	150 (82.9)	33 (47.8)	183 (73.2)	
ESBL	9 (5.0)	13 (18.8)	22 (8.8)	
MDR	5 (2.8)	12 (17.4)	17 (6.8)	
XDR	7 (3.9)	6 (8.7)	13 (5.2)	
CRE	0 (0.0)	4 (5.8)	4 (1.6)	
MRSA	10 (5.5)	1 (1.4)	11 (4.4)	

Notes:

* Mean (SD);

a Independent *t*-test applied; normality and equal variance assumptions met;

b Pearson chi-square applied;

c Fisher exact test applied;

MRSA = methicillin-resistant *S. aureus*; CRE = carbapenem-resistant *Enterobacteriaceae*

**Table 6:** The baseline characteristics associated with the survival status in patients with hospital-acquired BSI admitted to HPUSM using SLR (n = 250)

Variable	b	Crude OR (95% CI)	Wald statistics	*p*-value
Age (years)	0.05	1.06 (1.03, 1.08)	16.33	< 0.001

Gender				
Female	0	1		
Male	0.53	1.69 (0.94, 3.06)	3.04	0.081

Weight (kg)	−0.001	0.10 (0.98, 1.021)	0.01	0.910

Height (cm)	0.01	1.01 (0.97, 1.06)	0.24	0.625

Onset				
< 3 days	0	1		
3–7 days	−0.39	0.68 (0.19, 2.38)	0.37	0.545
> 7 days	−0.12	0.89 (0.25, 3.19)	0.04	0.852

Admission				
Ward	0	1		
ICU	0.37	1.44 (0.80, 2.60)	1.51	0.220

Department				
Surgical	0	1		
Medical	0.29	1.33 (0.76, 2.31)	1.01	0.316

APACHE score				
< 25	0	1		
> 25	1.31	3.70 (1.81, 7.54)	12.94	< 0.001

SOFA score				
< 9	0	1		
9–11	0.68	1.97 (0.67, 5.81)	1.511	0.219
> 11	1.82	6.16 (2.28, 16.65)	12.84	< 0.001

Diabetes mellitus				
No	0	1		
Yes	0.81	2.24 (1.09, 4.60)	4.85	0.028

Hypertension				
No	0	1		
Yes	1.01	2.747 (1.35, 5.61)	7.70	0.006

CKD				
No	0	1		
Yes	0.84	2.33 (1.29, 4.19)	7.93	0.005

COPD				
No	0	1		
Yes	1.05	2.87 (1.09, 7.56)	4.53	0.033

IHD				
No	0	1		
Yes	0.30	1.35 (0.52, 3.49)	0.38	0.540

CVA				
No	0	1		
Yes	0.81	2.24 (0.85, 5.94)	2.64	0.104

Malignancy				
No	0	1		
Yes	0.84	2.32 (0.92, 5.87)	3.15	0.076

Others				
No	0	1		
Yes	1.04	2.84 (1.02, 7.89)	3.99	0.046

Gram stain				
Negative	0	1		
Positive	0.02	1.02 (0.27, 3.87)	0.001	0.981
Fungal	0.37	1.44 (0.46, 4.57)	0.39	0.535

Strain				
Sensitive	0	1		
ESBL	1.88	6.57 (2.59, 16.64)	15.74	< 0.001
MDR	2.39	10.91 (3.60, 33.08)	17.83	< 0.001
XDR	1.36	3.90 (1.23, 12.35)	5.34	0.021
CRE	22.72	−0.46 (0.06, 3.68)	−0.55	> 0.950
MRSA	−0.79			0.460

Note: MRSA = methicillin-resistant *S. aureus*; CRE = carbapenem-resistant *Enterobacteriaceae*

**Table 7:** Associated factors of mortality in patients with hospital-acquired BSI admitted to HPUSM using multiple logistic regression (n = 250)

Variable	b	Adjusted OR (95% CI)	Wald statistics	*p*-value
Age	0.06	1.06 (1.03, 1.09)	14.11	< 0.001

Others				
No	0	1		
Yes	1.50	4.47 (1.32, 15.17)	5.76	0.016

Strain				
Sensitive	0	1		
ESBL	1.72	5.57 (2.04, 15.21)	11.24	0.001
MDR	2.29	14.70 (3.97, 54.83)	16.18	< 0.001
XDR	1.44	4.24 (1.08, 16.71)	4.26	0.039
CRE	22.42	−0.59 (0.07, 5.08)	−0.24	> 0.950
MRSA	−0.53			0.628

SOFA score				
< 9	0	1		
9–11	0.49	1.63 (0.48, 5.57)	0.61	0.436
> 11	1.43	4.16 (1.31, 13.19)	5.87	0.015

Notes: Forward LR Multiple Logistic Regression model was applied; Multicollinearity and interaction term were checked and not found; Hosmer-Lemeshow test (*p* = 0.344), classification table (overall correctly classified percentage = 80.0%) and area under the ROC curve (70.0%) were applied to check the model fit; MRSA = methicillin-resistant *S. aureus*; CRE = carbapenem-resistant *Enterobacteriaceae*
